# Postbiotics: An Alternative for Improving Health and Performance of Poultry Production

**DOI:** 10.3390/microorganisms13071472

**Published:** 2025-06-25

**Authors:** Fabricia Roque, Mário Henrique Scapin Lopes, Paulo Raffi, Ricardo Oliveira, Márcio Caparroz, Giovana Longhini, Carlos Granghelli, Douglas Faria, Lúcio Araujo, Cristiane Araujo

**Affiliations:** 1 Department of Animal Sciences, School of Animal Science and Food Engineering, University of Sao Paulo, Pirassununga 13635-900, Brazil; jotapvargas@gmail.com (F.R.); mhsl.br@usp.br (M.H.S.L.); giovanamachado@usp.br (G.L.); carlosgranghelli@usp.br (C.G.); defaria@usp.br (D.F.); lfaraujo@usp.br (L.A.); 2 Bender & Raffi Veterinary Services LTDA, São Paulo 15085-020, Brazil; pauloraffi@me.com; 3 Quiron Communication, São Paulo 02020-070, Brazil; ricardo@quironcomunicacao.com.br; 4 Agri BR-Agribusiness Strategy Consulting Services, São Paulo 17582-166, Brazil; mclp08@gmail.com; 5 Department of Nutrition and Animal Production, School of Veterinary Medicine and Animal Science, University of Sao Paulo, Pirassununga 13635-900, Brazil

**Keywords:** antioxidant, broiler, eubiosis, intestinal health, intestinal inflammation, postbiotics

## Abstract

The gastrointestinal tract has the largest surface area of the body and if it is subjected to continuous stress, it will activate the immune system. The intestinal barrier, the immune system, and the microbiota together form a line of defense against infection in order to maintain a healthy balance between the host and the microbiota. Nutritional strategies such as feed additives can be applied to maintain homeostasis in the gastrointestinal tract. When this equilibrium is disrupted, metabolic dysfunctions can occur. Postbiotics—the metabolic and/or structural compounds of microorganisms—can stimulate anti-inflammatory, antioxidant, immunomodulatory, and enzymatic effects, providing a safe alternative to establishing intestinal eubiosis. In addition, postbiotics can be used as an alternative to antibiotics in broiler diets by maintaining productive performance under challenging conditions. This review provides an overview of the postbiotic concept, mechanism of action and production, the evidence for the action in the gastrointestinal tract, and applications in poultry production.

## 1. Introduction

The gastrointestinal tract is a dynamic and complex environment that requires a correlation between the gut microbiota and the host for good functionality [1,2,3]. The composition of the gut microbiota changes throughout the life of the animal or due to various factors like the environment, behavior, and diets [4]. Studies indicate a strong correlation between intestinal diseases and the constitution of the microbiota [1,2].

A healthy gut is an essential condition for good poultry performance. Approximately 100 trillion bacteria colonize the gut [5] over an area of 200 to 300 m^2^ that enables diverse compositions and modulations of the gut microbiota. The barrier becomes unprotected and has less competition, resistance, and immune defense against the invasion of pathogens when the microbiota is reduced [6,7,8].

Over the past 40 years, a great number of scientific and clinical researchers have been studying the effects of probiotics, prebiotics, and symbiotics in the diet, due to the ability to positively modulate the microbial composition and establish intestinal balance [9]. Thus, pharmaceutical, food, and feed industries began using “biotics” in formulations and in commercial food additive-based products [10]. Since 2004, researchers have introduced a new concept of “biotic”—postbiotics. The International Scientific Association for Probiotics and Prebiotics (ISAPP) characterized the concept of a postbiotic in [10]. Studies have shown that postbiotics modulate the inhibitory activity to pathogens in gut microbiota, as well as the antioxidant activity exhibited by postbiotics, becoming a possible alternative as a zootechnical additive in animal production [11,12,13,14].

In addition, the use of postbiotics is economically feasible due to their long shelf life and no requirement for refrigerated storage [15,16]. Moreover, postbiotics have bacteriostatic and bactericidal capacity that decreases the pathogenic bacterial load in the gastrointestinal microbiota [17]. Since the intestinal microbiota and its microenvironment interactions plays a crucial role in poultry nutrition and overall health, postbiotics could possibly help to mitigate some of the most commons problems that revolve around gut health, such as high inflammatory status, high pathogenic bacteria colonization, and low antioxidant capacity, as well as challenging sanitary conditions, some of the factors that directly impact on birds’ productive performance and immunological status, while reducing antimicrobial usage in poultry farming.

Some major factors highlight important reasons for the use of postbiotics in poultry farming instead of common feed additives. Primarily, the main bird species in poultry (chickens, turkeys and ducks) present different pathogen challenges when compared to domestic mammals, being *Salmonela* spp., *Eimeria* spp., and *Clostridium perfringes* the most common and economically injuring pathogens in poultry, as well as differences in the immune system of birds, such as cell types, immunoglobulins and mucous layer [REF]. The use of postbiotics allows specificity in the mechanisms to control and mitigate infection by those pathogens, since the modulation of both the microbiome and its relationship with the immune system is a major mode of action of postbiotic products. This type of specificity in the direct combat of pathogenic infection is not obtained from other potential feed additives that possess plain anti-inflammatory and/or antimicrobial properties, such as some phytogenic compounds, essential oils, and acidifiers. In addition, postbiotics have a greater potential than directly fed microbials (DFMs) in mitigating pathogenic load while also being an alternative to growth-promoting antibiotics. Probiotics/DFMs use has been reported as being associated with the generation of antimicrobial resistance mechanisms by some researchers, since bacteria are able to transfer genetic material to each other by many different pathways. Probiotics and commensal microbes have characteristics that allow them to successfully replicate and colonize the GI tract of birds. Those certain traits, however, can be transferred to pathogenic bacteria also present in the same microenvironment through the known horizontal gene transfer mechanisms, such as genes related to antibiotic resistance mechanisms [18], meaning that the use of probiotics as an alternative to AGP may not be as effective as postbiotics, as there is a higher risk of promoting microbial resistance, the main issue that leads to AGP restrictions worldwide.

Thus, understanding how postbiotics act allows informed choices to be made regarding their use for clinical and/or commercial purposes, helping to provide further evidence that elucidates their mechanisms of action and facilitates decision making.

## 2. Postbiotics: Understanding the Concept and the Mechanisms of Action

Supplementing feed with probiotics, prebiotics, symbiotics, and postbiotics results in some positive effects on animal health (Table 1).

Probiotics require a proper concentration of live microorganisms during shelf life. However, in a fresh culture of the strain, living cells coexist with non-living cells. On the other hand, these non-living cells may be feasible, promoting a satisfying outcome in poultry production [19].

Postbiotic is a term derived from the words “biotic”, meaning “relating to or resulting from living organisms”, and “post”, a prefix for “after”. Since 2004, studies about the concept that non-living components of microorganisms promote the health and well-being of humans and animals have been gaining attention in scientific research. The literature reports a few synonyms for the term postbiotic, although in different forms: inactivated/non-viable microbial cells, heat-killed probiotics, paraprobiotics, ghost probiotics, metabiotics, tyndallized probiotics, and bacterial lysates [11,20]. A simple way to conceptualize the word: postbiotic means non-viable microbes and/or cell components with or without metabolites.

However, not every inactive and/or dead microorganism in food makes a postbiotic food, even though some strains can be used in both categories [18]. Postbiotics are metabolites and/or components derived from microorganisms, but a postbiotic is not necessarily derived from a probiotic. Inactivated microbial cells and/or metabolites or cellular components are present in the final product, but the viable cells are absent or insignificant. Evidence of a health benefit for the target host is required, as well as an evaluation of the safety in preparation for the intended use. Viruses, including bacteriophages, vaccines, filtrates without cellular components, purified microbial components (e.g., proteins, peptides, exopolysaccharides), and purified microbial metabolites (e.g., organic acids) are not considered postbiotics [11].

**Figure 1:**
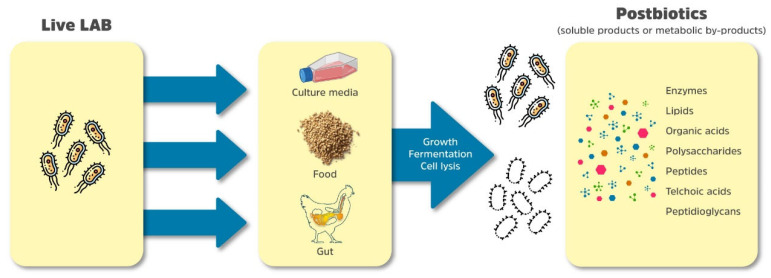
Schematic illustration for postbiotics concept from lactic acid bacteria in microbiological culture media, food and gastrointestinal tract [21].

Chemical and mechanical techniques are applied to prepare solutions in order to obtain postbiotics. Those processes promote the release of metabolic products after cell lysis, or during growth and fermentation, as in the case of postbiotics prepared from lactic acid bacteria that contain hundreds of individual metabolites used as a crude concentrate (extract) or as a semi-purified form, as in Figure 1 [21].

The amount and type of products are mainly related to the type of bacterial strain, type of culture medium, and bacterial treatment after propagation. However, the technique used to break the cell wall after lysis includes enzymatic extraction, solvent extraction, sonification, and heat [8,21]. The characterization of the progenitor microorganism (e.g., an annotated genome sequence) and description of the inactivation of the microorganism, procedure, and matrix must be provided for the qualification of postbiotic preparation. Furthermore, evidence of health benefits for the host presented in a research study and a report about the composition and evaluation of the safety in the target host for the intended use must be provided [11].

Postbiotics may contain intact inanimate microbial cells and/or microbial cell fragments/structures with or without metabolites/end products [11]. Thus, there are a number of microbial metabolites and/or microbial components, which can be classified as postbiotics. Microbial metabolites can be as diverse as enzymes, proteins/peptides, polysaccharides, organic acids, and lipids. Moreover, microbial components are obtained from live microorganisms, or released after microbial lysis, such as enzymes, peptides, teichoic acids, peptidoglycan-derived, muropeptides, polysaccharides, cell surface proteins, and organic acids [22,23].

Postbiotics support the gut immune function and modulate the microbiome [24]. The dosage level must be defined to measure the performance of a product in a specific action. However, most studies on postbiotics have not yet fixed a specific animal dose [25]. In contrast, the use of postbiotics in the diet of production animals indicates satisfactory results in immunomodulation, antimicrobial, anti-cancerous, antioxidant, and anti-diabetic actions, as well as a reduction in food allergies [26].

Although the exact mechanism underlying the action of postbiotics is still unknown, scientific data provide evidence of the role postbiotics play in various physiological functions. In addition, the classification of postbiotics can be based on composition or physiological functions, as follows.

Physiological functions of postbiotics are listed below:

- Immunomodulation: postbiotics promote selectivity, cytotoxicity against tumor cells, protection of the intestinal epithelium through apoptosis of normal epithelial cells, and improvement in IgA, IFN-γ, and IL-10 secretion [27]. In addition, they induce differentiation of T lymphocytes to establish the balance between Th1 and Th2 lymphocytes [8].
- Prevention of intestinal infection: postbiotics act as an intestinal barrier by altering the expression of host genes or modulating the local environment [28]. Also, they exercise the inactivation of pathogens through the production of short-chain organic acids and/or antimicrobial peptides, causing antagonistic intervention with pathogens by adhering to intestinal epithelial cells and promoting the production of IgA [29].
- Oxidative stress: postbiotics display antioxidant and anti-inflammatory actions to combat diseases that affect the gastrointestinal tract [12].

The mode of action capable of improving host health is not completely comprehended yet. Postbiotics can stimulate and/or modulate the host immune response, involving both the acquired immune response and the acquired host response [30]. This fact suggests the initial response is related to the innate immune system and is composed of a number of pattern recognition receptors capable of interacting with microorganisms. The nucleotides-binding and oligomerization domain (NOD)-like receptors (NLRs) and the toll-like receptors (TLRs) are two of the pattern recognition receptors implicated in the host response to postbiotics [25]. Nevertheless, the exact immune system activation pathways and interactions that are promoted by these types of molecular signaling are still not fully understood, nor how each different postbiotic compound can regulate or interact with immune mechanisms such as the receptors mentioned.

## 3. Postbiotics: Methods of Production and the Main Compounds

Most bacteria have the capacity to synthesize polysaccharides. Microorganisms can produce peptidoglycan and lipoteichoic acids—the structural polysaccharides found in Gram-positive bacteria cell walls—as well as glycogen, located in the cytoplasm, and lipopolysaccharides, anchored in the Gram-negative bacteria outer membrane [31].

Earlier studies have reported that the most frequent postbiotic components are the cell wall fragments [8,32], cell-free supernatants [33,34], short-chain fatty acids [35], bacterial lysates [36], and metabolites.

**Figure 2:**
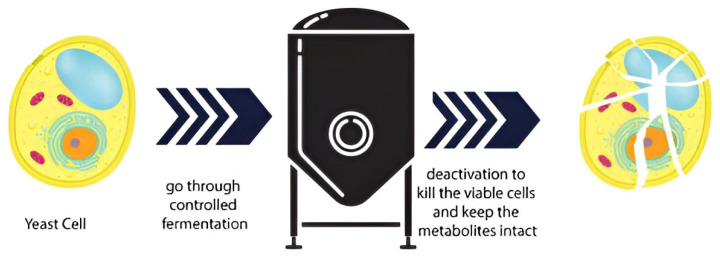
Postbiotics are obtained through live (viable) microorganisms that are subjected to microbial action—such as fermentation—to produce their active metabolites and then are deactivated to turn such microorganisms inanimate.

Cell wall fragments are either components of the bacterial cell wall of Gram-positive bacteria or, mainly, components of yeast cell wall and are characterized as immunogenic [8]. In some Gram-positive bacteria, cell wall fragments serve as messengers in bacterial communication and associate molecules in spore resuscitation and germination [37]. Gram-positive bacteria produce compounds that are advantageous to the host, such as peptidoglycan, teichoic acids, polysaccharides, and proteins [32]. The peptidoglycan (PG), a polymer structure of some microorganisms’ cell walls, is necessary for preserving the cell shape and survival [38], also playing a significant role in signaling innate immunity, organ development, and behavior. Cell-free supernatants contain biologically active metabolites secreted by bacteria and yeasts. The bacteria and/or yeasts undergo a period of incubation, and then the microbes are centrifuged and removed. Lastly, the final product is filtered to ensure sterility (Figure 2).

*Lactobacillus acidophilus* and *Lactobacillus casei* supernatants from probiotic strains show good anti-inflammatory and antioxidant properties, and act on intestinal epithelial cells and then on immune cells. However, immunomodulatory action depends on the source of the probiotic strain, and in vivo studies should be conducted on this matter [33].

The gut microbiota ferments starch, the non-starch polysaccharides, and the dietary fiber producing the short-chain fatty acids (SCFAs). The composition of the SCFA (acetate, butyrate, or propionic) depends on the substrates and the microorganisms that digest these substrates. Butyrate acts as immunosuppressive, an energy source for the enterocytes, renewal of the intestinal epithelium, and modulation of gene expression [38,39]. Propionic acid has a protective action that prevents the colonization of pathogenic bacteria in the intestinal lumen [35]. Bacterial lysates are obtained by chemical or mechanical degradation of Gram-positive and negative bacteria found in the environment [8]. The method is very advantageous due to not having protein denaturation during the mechanical disruption of cells, besides the characteristic of improving host defense against pathogens, preventing respiratory and intestinal infections, and subsequent inflammatory conditions [36]. The gut microbiota produces an array of molecules, including vitamins, phenolic-derived metabolites, and aromatic amino acids. Due to high bioavailability, antioxidative features, and signaling properties, these substances are considered important contributors to host–microbiome crosstalk [8].

Postbiotics derived from the fermentation process of vitamins and enzymes can promote intestinal health and host nutrient digestion and absorption, and act on villous growth and immunity [13]. Microorganisms in the growth phase will produce biopolymers with different chemical properties. These biopolymers can be released outside the bacterial cell wall, forming a heterogeneous group of substances called extracellular polysaccharides (EPs). EPs have also gained more attention in food applications due to the interaction with dendritic cells (DCs) and macrophages that modulate the immune response. In addition, EPs increase the proliferation of T lymphocytes and NK cells [40], increase IgA concentrations in the intestinal mucosa, and stimulate lymphocyte proliferation [41], showing the antimicrobial and antioxidant actions of this kind of postbiotic, as seen in Table 2 [42].

The type of microorganism, culture medium, and post-propagation treatment of the microbe, among other factors, could also influence how the postbiotics behave within the organism [8,21,32]. The mechanism of action may operate individually or in concert [11]. However, more research on extraction protocols and analytical tools to detect, identify, and quantify postbiotics is still needed. The use of metabolomics enables the accurate measurement of small molecules in a complicated biological system [23].

## 4. Eubiosis and Gut Health: Microbiota and Gut Barrier

The intestinal ecosystem is composed of microbiota, intestinal barrier, and immune system, and its main function is to keep the interaction between host and microbiota in balance. Thus, the microbiota is constantly changing in the microbial profile of the host and continuously challenges these interactions [43]. The gut microbiota is diverse, complex, and constantly changing [44]. Five major phyla reside in the gastrointestinal tract [45], as phylum Proteobacteria (*Escherichia, Helicobacter*), Firmicutes (*Bacillus* spp.), Actinobacteria (*Bifidobacterium* spp.), and Bacteroidetes (*Bacteroides* spp.), that represent the majority of bacterial species, approximately 90% [46]. Bacterial growth is dependent on the availability of nutrients. Bacteria extract, synthesize, and absorb nutrients and metabolites from bile acids, lipids, amino acids, vitamins, and short-chain fatty acids (SCFA) to grow and reproduce [10,46].

**Figure 3:**
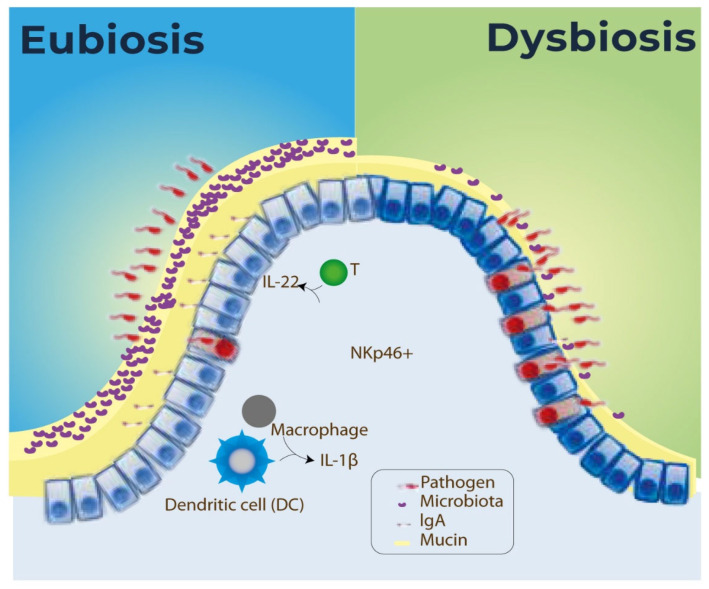
Defenses of microbiota in eubiosis or in dysbiosis [6].

The bacteria that form the intestinal microbiota can be widely categorized as commensal or pathogenic. Commensal bacteria live in the body and confer some benefits, while pathogenic bacteria cause disease in certain conditions. Dysbiosis occurs when the gut bacteria suffer abnormal changes in commensal or pathogenic bacteria quantity or species, as exemplified by Figure 3 [47,48].

The intestinal microbiota provides three levels of protection against pathogen action. In the first protection zone, the microbiota competes with pathogens for colonization, and nutrients for barrier maintenance and immune defense. In the case of dysbiosis, the resistance is lower and the propensity for pathogen attack is higher [6]. Previous studies have shown the beneficial effects of postbiotics in controlling pathogens. For example, the postbiotic tested in vitro through the agar-disk diffusion method and the postbiotic synthesized by *Lactobacillus salivarius* cell supernatant showed promising antibacterial activity on the pathogen (*Escherichia coli*). The beef analysis with different concentrations of postbiotic conferred the minimum effective amount of 35.00 mg/g against the pathogen *E. coli* [49].

The intestinal barrier is composed of epithelial cells that are exposed to microbes in the lumen. The intestinal barrier function, also called intestinal integrity, provides physical barriers against the invasion of intestinal microorganisms [50]. In addition, the calyceal cells secrete mucins that generate a layer of mucus in the form of a gel structure in the intestinal mucosa. The mucus layer and the epithelial cells form two protective layers. The internal layer is firmly adherent to the intestinal epithelium, is free of microorganisms, and acts against adhesion and invasion of pathogens. The external layer is inhabited by microorganisms and formed by proteolytic and glycosidic degradation of highly polymerized gel and mucin, secreted by the host or the bacteria [7]. The intestinal barrier refers to a line of defense against the action of pathogens, rather than a physical structure. This way, nutrient exchange and immune defense happens according to flows of two different routes: transcellular and paracellular. The transcellular pathway is responsible for the absorption and transport of nutrients through specific channels or pumps. The paracellular lane is responsible for the transport between cells, over cell junctions [51].

The action of pathogens occurs through cell junctions and can be direct, through proteins, or indirect, through phosphorylation or dephosphorylation of proteins. The immune system can also influence the permeability of the intestinal barrier [51]. The innate immune system and the adaptive immune system compose the immune system. The first line of defense against pathogens is the innate system. Antigen-presenting cells (APCs), a diverse collection of immune cells that mediate the cellular immune response by processing and presenting antigens for detection through specialized lymphocytes, such as T cells, are the agents of the adaptive system, a non-specific innate response. Through innate immunity and the structure of the intestinal barrier, the immune system protects the body from pathogen attacks. The T cells found in the intestinal mucosa help to maintain the eubiosis condition in the digestive tract while defending against infections. The way gut bacteria can specifically target mucosal T lymphocytes is still unclear [51]. The immune cells need energy to carry out functions like phagocytosis, activation, antigen presentation/processing, migration, phosphorylation, differentiation, and effector responses [52,53].

The immune system controls the exposure of bacteria by reducing the action of pathogens through stratification and compartmentalization. Stratification minimizes the direct contact between the intestinal bacteria and the epithelial cell surface, while compartmentalization confines the bacteria to intestinal sites and limits exposure [44]. Stratification of gut bacteria secretes immunoglobulin A (IgA), an intestinal bacteria produced from intestinal dendritic cells. These dendritic cells are loaded with bacteria that interact with B and T cells in Peyer’s patches and induce the B cells to produce IgA to fight the intestinal bacteria. B cells shelter in the intestinal lamina and secrete IgA, later permeated through the epithelium, and deposited on the apical surface. The permeated IgAs are bound to luminal bacteria and prevent microbial translocation across the epithelial barrier [54]. Furthermore, postbiotics provide protection against enteric infections caused by pathogens in the mucosal areas of the GI tract by increasing IgA levels [34,55].

## 5. Reported Impacts on Productivity and Poultry Health

A healthy gut is an essential condition for production animals to perform well, and even more prominent when considering antimicrobial restrictions in livestock production worldwide [56]. Studies corroborate the positive effect of using postbiotics on the performance of broilers, as seen in Table 3.

For example, birds fed with 10% phytase co-fermented wheat bran (FWB) alongside with 10^8^ *Saccharomyces cerevisiae* had significantly greater body weight (BW), with an average of 873 g/bird (10% FWB) vs. 797 g/bird (control) and greater body weight gain (BWG), approximately 824 g/bird (10% FWB) vs. 748 g/bird (control) during the period 1–21 days of age. FWB supplementation also improved villus height, increased the numbers *of Lactobacillus* spp. in the cecum, and increased ash content in the femur. This ash indicates that postbiotic supplementation improved intestinal health characteristics and bone mineral concentration in broiler chickens. The postbiotic further enhanced immune response and anti-inflammatory capacity through epigenetic mechanisms of mRNA expression of pro-inflammatory cytokines, including interleukin-6 (IL-6), nuclear factor-κB, and IL-1β. This factor was lower in chickens supplemented with 5% and 10% of postbiotic compared to the control [13].

Pascual and coworkers [57] reported that postbiotic supplementation based on yeast cell wall extract did not affect final body weight. Despite this, the supplementation decreased feed intake, improved feed conversion, increased villus height and the number of goblet cells. The density of intestinal inflammatory cells was also reduced (CD45+) when under the challenging conditions of intensive rearing systems in broiler chickens. Thus, the use of postbiotics in broilers improves intestinal health characteristics due to the increased villus height, absorptive area, and improved villus-height-to-crypt-depth ratio [17,56,57,59,60].

### 5.1. Antioxidant Action and Meat Quality Interactions

Not only do postbiotics interact with immune components in birds but they also modulate antioxidant features. Lin and colleagues [58] reported antioxidant activity in broilers supplemented with the postbiotic by *Laetiporus sulphureus* fermented product during a period of 35 days of age. The study reported a significant increase in the activity of superoxide dismutase (SOD) and, at the same time, decreases in the concentration of malondialdehyde. These findings justify the effect of antioxidant properties of the tested postbiotic in broiler meat. Likewise, up-regulation of gene expression of the nuclear factor erythroid 2–related factor 2 (*Nrf2*), as well as antioxidant enzymes such as heme oxygenase-1 (HO-1) and SOD genes in the liver and jejunum, were also reported. Possibly, this further indicates the antioxidant role of postbiotics in broilers. Similarly, [12] observed that the supplementation of postbiotic by *Lactobacillus plantarum* RI11 increased plasma activities in total antioxidant capacity, catalase and glutathione, while reducing the decrease in α-1-acid-glycoprotein (1-AGP) and ceruloplasmin (CPN) in heat-stressed broilers. This aspect indicates that postbiotic dietary inclusion favors the improvement of the oxidative capacity of broilers, thus reducing malonaldehyde in broiler meat, essentially demonstrating the benefits of postbiotics to mitigate lipid peroxidation during the growth phase of poultry, even under heat challenges. Furthermore, postbiotic supplementation can affect meat quality beyond lipid peroxidation. In an experimental trial, broilers fed various levels of *S. cerevisiae* fermentation product showed increases in carcass weight and leg yield [66]. This report suggests that postbiotic inclusion could improve nutrient absorption and digestion, including minerals and vitamins. Different amounts of postbiotics altered the pH and the level of oxidative lipolysis in the animals, indicating that the changes in the meat quality may be related to the anti-inflammatory and antioxidant properties.

### 5.2. Intestinal Morphology and Performance Enhance

Thanh and coworkers [59] observed that a blend of metabolites produced by *L. plantarum* strains (RS5, RI11, RG14, and RG11) increased final BW, BWG, and average daily weight gain, and decreased FC. The research found improvements in gut health characteristics, such as the increase in lactic acid bacteria population, small intestine villus height, and fecal volatile fatty acid population. Similarly, [67] evaluated different dosages of postbiotic combinations produced from three strains by *L. plantarum* (RI11, RG14, and RG11). The analysis demonstrated that 0.6% of the supplementation from this combination significantly increased egg production and fecal lactic acid bacteria population while also reducing fecal pH and Enterobacteriacea count. In addition to these observations, small intestinal villus height increases were observed. An interesting effect of the supplementation was also observed regarding reductions in plasma and yolk cholesterol concentrations in early commercial layers during 19 to 31 weeks of age. This effect can be relevant in terms of reducing abdominal fat, allowing for better laying consistency, while also promoting eggs with lower cholesterol for consumers.

The synergistic effects of postbiotic and prebiotic supplementation on broiler production rates are significant. For instance, a study with different postbiotics synthesized by lactic acid bacteria and inulin levels showed significant increases in final weight of the birds (2334.90 g/bird in 0.3% RI11 + 0.8% inulin-fed birds vs. 2239.59 g/bird negative control), increases in weight gain by 2281.31 g/bird (0.3% RI11 + 0.8% inulin) **vs.** 2189.10 g/bird (NC), and a decrease in FC from 1.84 points (0.3% RI11 + 0.8% Inulin) vs. 1.94 points (NC). In addition, villus height in the duodenum and ileum and the fecal lactic acid bacteria increased, while the fecal pH and Gram-negative bacteria count (*Enterobacteriaceae*) decreased when compared to the negative control. The results indicate the supplementation of postbiotic and prebiotic in broiler diets generates a synergistic effect [17]. Similarly, [60] found that supplementation by fermented soybean meal with or without mannan-oligosaccharides increased weight gain but reduced feed conversion and plasma 3-methylhistidine concentration. Furthermore, this dietary inclusion increased villus height and the ratio of villus height to crypt depth in the duodenum, increased lactic acid bacteria (fecal, ileal, and cecal), reduced pH (fecal, ileal, and cecal), and *Clostridium perfringens* (ileal and cecal) compared to the control diet, indicating synergistic effects and a fermentation process by combining fungal and bacterial species.

The bacteriostatic and bactericidal capacity present in the postbiotics decreases the pathogenic bacterial load in the gastrointestinal microbiota [17]. For example, studies developed on rats investigated the ability of *S. cerevisiae* and *Saccharomyces boulardi* yeast postbiotics to reverse or treat intestinal discomfort caused by acute stress, and the results revealed an improvement in small and large intestinal characteristics and restoration of motility [68]. Similarly, Takadanohara and colleagues [69] reported that postbiotics supplementation with microbial lysates improved the characteristics of villus height, crypt depth, the number of goblet cells in the villi and crypt, besides the prevention on the intestinal barrier, in the jejunum and in the ileum under chronic stress in rats. Likewise, similar responses could be observed in poultry, as reported by [62], where broilers supplemented with yeast fermentate had lower cortisol concentrations in response to the acute stress of 1156.58 pg/mL (stressed 1.6% postbiotic group) vs. 1807.68 pg/mL (stressed control) and lower heterophil/lymphocyte ratios in response to chronic stress of 0.5 pg/mL (stressed 1.6% postbiotic group) vs. 0.68 pg/mL (stressed control). This suggests that yeast fermentate supplementation reduces both short and long-term measures of stress.

### 5.3. Immune System Modulation and Pathogen Control

Epithelial cells and the intestinal barrier protect the host from the attack of pathogens. Dietary nutrient products from the gastrointestinal tract and medications can break the epithelial barrier, leading to the shedding of epithelial cells and the development of wounds [70]. In poultry production, the association of bacteria and the egg industry is extremely important due to the possible risk to public health, especially with regard to the foodborne pathogen *Salmonella* spp., belonging to the family Enterobacteriaceae. Salmonella contamination can occur through eggshells or the reproductive tract, and infection in the layers of ovaries via systemic infection, or through contaminated cloaca [71].

Postbiotic supplementation can also enable the restitution of intestinal cells. For example, in vitro, *S. boulardii* supernatant supplementation increased the restitution of differentiated epithelial cells and enhanced enterocyte migration but did not influence proliferation, suggesting that wound healing was associated with increased cell motility rather than stimulation of cell proliferation [72]. In addition, adhesion assays of *S. boulardii* supernatant supplementation modulated the α2b1-dependent cell interaction with type I collagen, indicating that *S. boulardii* can modulate pathways leading to cell migration [72].

Additionally, postbiotics can be used to treat *Salmonella* [25] and can reduce the amounts of pathogens in broilers and layers. In [14], it was reported that layer pullets challenged with *Salmonella* Enteritidis (SE) at 106 CFU/mL and supplemented with *S. cerevisiae* fermentation-based postbiotic reduced the SE colonization load by 4.49–3.35 log CFU/gram of ceca when compared to the positive control. This evidence indicates that the postbiotic provided an improved intestinal microflora and a hostile environment for the SE to colonize, implying a better bacterial load. Considering the immature immune system in pullets and the long production cycle, the capacity of mitigating SE colonization may provide long-term effects regarding future hen gut health and laying performance. In [41], it was reported that chicks challenged with *Salmonella* Typhimurium and fed *L. plantarum* LTC-113 had lower colonization of *S.* Typhimurium in the liver, spleen, and ceca (*p* < 0.05) and a lower intestinal permeability compared to the animals that did not receive the postbiotic. This indicates that there was Salmonella infection due to the loss of epithelial integrity, as indicated by increased permeability and by supplementing with *L. plantarum* LTC-113, denoting that the damage of intestinal morphology reduced and decreased inflammation scores. Researchers in [62] reported that laying hens fed postbiotic *S. cerevisiae* fermentation-based metabolites of Diamond V Original XPC™ diets displayed increased BW at 12 weeks of age. The dietary treatment did not significantly affect the relative weights of the ovary, bursa, and ceca, but the challenge with *Mycoplasma gallisepticum* had a significant effect on the relative weights of these organs at 12 weeks of age. The scientists also stated that birds exposed to *Mycoplasma gallisepticum* contracted the disease and had an immunological reaction as a result.

Previous research has demonstrated that postbiotics can be a viable alternative to antibiotics. Postbiotics have a preventative and restorative mechanism in the gastrointestinal tract and may alter the gastrointestinal microbiota, with a reduction in the pathogenic bacterial load. Teck and colleagues [64] reported that broilers fed a combination of four strains of *L. plantarum*—RS5, RI11, RG14, and RG11—displayed a reduced number of Enterobacteriaceae and an increased number of lactic acid bacteria, probably due to the lower fecal pH. The authors also reported the effect of postbiotic supplementation in broiler diets may be an addition to zootechnical characteristics and intestinal health. Supplementation of the postbiotic combination can reduce the concentration of cholesterol in broilers, as observed in the disaggregation of bile acids that corroborate in order to identify the removal of cholesterol from the body. In addition, the report of an increase in the capacity of bile acids disaggregation and a reduction in cholesterol concentrations in plasma and meat compared to the control indicates that postbiotic supplementation favors the reduction in cholesterol in broilers. Distinctively, in broiler chicken, lower plasma cholesterol concentrations are more desired than other poultry categories, since they are associated with less abdominal fat in carcasses, being lean meat more desired by customers [73].

Several factors can cause the destabilization of gut microbiota. Heat stress is one of the conditions that makes the animal susceptible to the attack of pathogens [74]. “Heat shock proteins” (HSP70) is one of the basic mechanisms of cell defense and protection under stress conditions, whereas the acute phase proteins (APPs) restore homeostasis and prevent microbial proliferation after stimulation of innate immunity [75,76]. HSP70 and APPs are proteins that signal the animal’s heat stress conditions. Therefore, an increase in the concentration of HSP70 indicates that the animal was exposed to thermal stress and therefore susceptible to reduction in productive performance. The study by [71] about supplementation of different postbiotics in broilers subjected to heat stress had an inconsistent result and no significant difference for HSP70. The supplementation of 0.3% postbiotic *L. plantarum* RI11 reduced the concentration of APPs and enhanced the immune response in a way to neutralize the negative effect of heat stress on broilers. Kaufman and coworkers [75] reported the beneficial effects of postbiotic supplementation from Aspergillus oryzae on the productive performance of dairy cows under chronic stress. The authors observed a reduction in the concentration of APPs in cows supplemented with 3 g/day of postbiotic when compared to control cows, indicating that postbiotic supplementation enabled the cows to resist heat stress.

In contrast, Price and coworkers [65] reported that HSP70 was not affected in broilers fed with metabolites of Diamond V Original XPC™. However, heat stress significantly increased the plasma HSP70 concentrations when compared to no heat stress. Moreover, supplementation reduced the concentration of plasma corticosterone and improved the heterophil/lymphocyte ratio and composite asymmetry score in comparison with control in heat stress or during normal rearing conditions at 42 days of age. This suggests that postbiotics may have an impact on animal health and well-being, despite further research being required to completely comprehend the mechanisms affecting both the immune system and the stress response.

Furthermore, postbiotic efficiency has been reported as closely associated with diet composition. In a study with a nutritional-deficient diet, containing high quantities of nonstarch polysaccharides (NSPs) due to barley and rye dietary inclusion, postbiotic supplementation provided an increase in BW at 35 days of age. However, in the standard, non-challenged diet, the inclusion of the postbiotics did not affect any of the main productive performance indexes [77]. The same group of researchers from the previous study also reported a diet composition influence regarding the cecal microbiome in broiler chicken, since the inclusion of postbiotics in a standard, non-challenged diet did not promote any significant changes in the microbiome, but an effect on short-chain fatty acid concentrations was reported when supplementing postbiotics in an NSP-rich diet. Moreover, regardless of the diet composition, postbiotic supplementation did not affect any of the immune status parameters evaluated [78]. In a study by Forder and coworkers [79], the dietary supplementation of *Saccharomyces cerevisiae* postbiotic for two genetic great grandparents’ lineages did not influence intestinal permeability parameters, corticosterone concentrations, or white blood cell concentration.

A wide range of probiotics used in poultry have been reported in the literature in the past years, while also presenting a potential source for postbiotic production. Therefore, studies that report intestinal health effects due to probiotic use will be discussed in this section. Probiotics are used in livestock production mainly as a way to enhance gut health, allowing for stable growth and animal performance, encompassing many bacterial species used with interesting results, usually due to a combination of different mechanisms of action from the probiotic products [80]. For instance, the use of a multi-strain *Bacillus* compound in the diet of *C. perfringens*-challenged broilers resulted in increased duodenal villi height, as well as increased villi height/crypt ratio in both duodenum and jejunum when compared to un-supplemented birds, as well as an improvement in BW and FCR of birds supplemented with the feed additive [81]. This enhancement in bird performance, even when broilers were fed diets with reduced metabolizable energy, containing high-NSP content feed ingredients such as maize and wheat bran, shows the efficacy of probiotic strains in enhancing digestibility, allowing for a more economic production, since feed cost can be reduced by the inclusion of those feed ingredients without compromising bird performance.

Probiotics can be extracted and isolated from the gut of healthy chickens and then used as feed additives for another flocks. In a study that involved both in vitro and in vivo trials, a multispecies probiotic product containing strains of *Enterococcus faecium*, *Pediococcus acidilactici*, *Lactobacillus salivarius*, *Lactobacillus reuteri*, and *Bifidobacterium animalis* were isolated from the GI tract of healthy chickens and administered via drinking water to day-old chicks, including chicks challenged with *Clostridium jejuni* [82]. The authors reported an expressive reduction in the cecal colonization by the pathogen, which leads to a reduction in chicken meat contamination and possible infection of consumers.

## 6. Future Perspectives

The capabilities of fast genome sequencing allowed in modern days are now able to allow a better understanding of concepts and interactions that were unexplained until now. The tracking of bacterial metabolites using high technology metabolomic analysis is a key factor in identifying metabolic pathways that are being enhanced or decreased by the use and interactions concerning postbiotics, mainly by providing a better understanding of the microbiota and host immune system interactions and how feed additives such as postbiotics are able to orchestrate such refined mechanism of control.

Nonetheless, with these molecular techniques, it will be possible to understand how each individual interacts with each type of bacteria species, and furthermore, with their metabolites, meaning that, in a few years, adequating the use of different feed additives to individuals with the most affinity or ability to make better use of that specific product is a possibility.

Therefore, the main goal of future research is to integrate both feed technology research, in a way that better control and definition of each compound is fully understood, and providing a full clarification of the role of each metabolite and its interaction within the host microbiota, in a way to refine poultry production even further.

## 7. Conclusions

In summary, supplementation of postbiotics can improve performance characteristics, intestinal health parameters, and overall oxidative capacity in diverse poultry species, as well as provide a higher count of beneficial bacteria to the microbiome environment and also enhance the restitution of intestinal cells while reducing intestinal inflammation, valuable characteristics that can improve performance and health in flocks. Likewise, the dietary inclusion of postbiotic combinations can have additive, beneficial effects for birds, such as the combination of postbiotics and phytase, enhancing the immune response and anti-inflammatory capacity through epigenetic mechanisms in the mRNA expression in pro-inflammatory cytokines. Furthermore, postbiotic supplementation can improve the total antioxidative capacity of broilers and therefore reduce the malonaldehyde content in meat, allowing for better carcass quality. However, despite the reported advantages of postbiotics that were brought in this review, further research is yet required to understand the more complex interactions between the compounds and their relationship within the diverse range of poultry organisms and their immune systems.

## Figures and Tables

**Table 1:** Definitions of “biotics”.

Terms ^1^	Definition
Probiotic	Live microorganisms, administered in adequate amounts: confer a health benefit on the host
Prebiotic	Substrate selectively utilized by host microorganisms conferring a health benefit
Symbiotic	A mixture comprising live microorganisms and substrate(s) selectively utilized by host microorganisms that confers a health benefit to the host
Postbiotic	Preparation of inanimate microorganisms and/or their components that confer a health benefit to the host
Parabiotic	Non-viable microbial cells, administered in adequate amounts: confer a benefit to the host

^1^ Source: [11].

**Table 2:** Main extracellular polysaccharides.

Exopolysaccharide (EP)	Original Bacteria	Potential Fields of Application	Reference
D-Glucose, D-Galactose, Mannose, Xylose	*Lactobacillus delbrueckii* ssp. bulgaricus (OLL1073R-1)	(Non-poultry) Fermented milk products, such as yogurt for human consumption	[40]
Glucose, Galactose	*Lactobacillus plantarum* JLK0142	Potential use as functional food or feed additive for macrophage immunomodulation against pathogens	[41]
D-Glucopyranose, D-Galactopyranose	*Lactococcus lactis* ssp. F-mou (LT898177.1)	Feed additive against pathogens, such as *E. coli* and *Lysteria monocytogenes*	[42]

**Table 3:** Effects of different postbiotic supplementation in poultry.

Postbiotics	Results	References
*Saccharomyces cerevisiae* and phytase co-fermented wheat bran	Birds fed with 10% wheat bran fermented alongside postbiotics showed higher BW, BWG, and FC, improved villus height and number of Lactobacillus spp. in the cecum, and lower expression of pro-inflammatory cytokines.	[13]
Yeast cell wall extracts	Postbiotic supplementation decreased feed intake and improved feed conversion, increased villus height and the number of goblet cells, and reduced the density of intestinal inflammatory cells.	[57]
*Laetiporus sulphureus* fermented product	The postbiotic supplementation increased the activity of superoxide dismutase (SOD) and at the same time decreased the concentration of malondialdehyde.	[58]
*Lactobacillus plantarum* RI11	The postbiotic supplementation increased plasma activities of total antioxidant capacity, catalase, and glutathione and reduced the decrease in α-1-acid-glycoprotein and ceruloplasmin in heat-stressed broilers.	[12]
*Blend of Lactobacillus plantarum* (RS5, RI11, RG14 e RG11)	Results indicate that the blend of probiotics increased final BW, BWG, average daily weight gain and lower FC, increased lactic acid bacteria population, small intestine villus height, and fecal volatile fatty acid population.	[59]
Metabolic products synthesized by lactic acid bacteria + Inulin	Results indicate the synergistic effect under probiotics + inulin increased the final BW, and reduced FC, increased villus height in duodenum and ileum and fecal lactic acid bacteria, and decreased fecal pH Enterobacteriaceae.	[17]
Fermented soybean meal with or without mannan-oligosaccharides	The use of a postbiotic with a prebiotic increased BWG, reduced FC and reduced plasma 3-methylhistidine concentration, increased villus height and the villus height to crypt depth ratio in the duodenum, increased lactic acid bacteria (fecal, ileal, and cecal), reduced pH (fecal, ileal and cecal) and reduced Clostridium perfringens (ileal and cecal).	[60]
*Pleurotus* *eryngii* stalk residues	Supplementation of 0.5% postbiotic increased body weight gain, the rate of lactic acid bacteria in relation to pathogens in the cecum, villus height in the ileum, and jejunum and the villus height/crypt depth ratio in birds at 35 days of age.	[61]
Fermented Yeast	Yeast fermented supplementation resulted in lower cortisol concentrations in response to acute stress and lower heterophil/lymphocyte ratios in response to chronic stress in broilers when compared to controls.	[62]
Saccharomyces cerevisiae fermentation-based metabolites	Reduced the amount of Salmonella Enteritidis colonization compared to the positive control	[14]
Lactobacillus plantarum LTC-113	Chicks challenged with Salmonella Typhimurium and fed Lactobacillus plantarum LTC-113 had less S. Typhimurium colonization in the liver, spleen, and caeca, and intestinal impermeability compared with the animals that did not receive the postbiotic	[41]
Saccharomyces cerevisiae fermentation-based metabolites	Dietary treatment of the birds did not significantly affect the weights of the ovaries, bursa, or cecum, but the challenge with Mycoplasma gallisepticum caused a significant impact on the weights at 12 weeks of age.	[63]
L. plantarum RS5, RI11, RG14 e RG11	Broilers fed a combination of postbiotics reduced the number of Enterobacteriaceae and increased the number of lactic acid bacteria, probably due to the lower fecal pH.	[64]
Saccharomyces cerevisiae fermentation-based metabolites	Blood corticosterone concentration increased significantly in the absence of heat stress and decreased in the presence of heat stress and improved heterophil/lymphocyte ratio and composite asymmetry score (0.54–1.50; *p* < 0.0001) compared to control and at 42 days of age under heat stress or during normal rearing conditions	[65]

## Data Availability

No new data were created or analyzed in this study.
